# Exploring WHO Communication during the COVID 19 Pandemic through the WHO Website Based on W3C Guidelines: Accessible for All?

**DOI:** 10.3390/ijerph17165663

**Published:** 2020-08-05

**Authors:** Elena Fernández-Díaz, Patricia P. Iglesias-Sánchez, Carmen Jambrino-Maldonado

**Affiliations:** Department of Economics and Business, University of Malaga, Av. de Cervantes, 2, 29016 Málaga, Spain; efernandezdiaz@uma.es (E.F.-D.); patricia.iglesias@uma.es (P.P.I.-S.)

**Keywords:** World Health Organization, web accessibility, WCAG 2.1, COVID-19, public health, pandemic, communication, W3C

## Abstract

Health crisis situations generate greater attention and dependence on reliable and truthful information from citizens, especially from those organisations that represent authority on the subject, such as the World Health Organization (WHO). In times of global pandemics such as COVID-19, the WHO message “health for all” takes on great communicative importance, especially from the point of view of the prevention of the disease and recommendations for action. Therefore, any communication must be understandable and accessible by all types of people, regardless of their technology, language, culture or disability (physical or mental), according to the World Wide Web Consortium (W3C), taking on special relevance for public health content. This study analysed whether the WHO is accessible in its digital version for all groups of citizens according to the widely accepted standards in the field of the Internet. The conclusion reached was that not all the information is accessible in accordance with the Web Content Accessibility Guidelines 2.1, which implies that there are groups that are, to some extent, left out, especially affecting the elderly. This study can contribute to the development of proposals and suggest ways in which to improve the accessibility of health content to groups especially vulnerable in this pandemic.

## 1. Introduction

The use of the Internet has increased in the last decade, as approximately 60% of the world’s population now uses this medium. In 2020, there are more than 4.5 billion people using the Internet [1]. Therefore, not only has the capacity of access to the Internet increased but more and more users have access to Internet content, also as a source of information.

In the face of a pandemic such as COVID-19, access to reliable information by citizens is crucial as a means of preventing the disease and enabling citizens to take action in certain everyday situations, as demonstrated in a study by the European Parliament’s European Science-Media Hub [2]. The World Health Organization (WHO) website is one of the main sources of information for the public, providing daily updates and interactive maps showing the evolution of the pandemic and offering credibility and security of information [2]. In addition to the WHO, other international sources of information stand out, such as the European Centre for Disease Prevention and Control and the European Commission’s Coronavirus and Coronavirus Response page, as well as the European Medicines Agency, among others.

As has happened in the world with the Zika and Ebola pandemics, the Internet is not only a source of communication for prevention and action in the face of health crises but also becomes a channel for misinformation [3,4], making the need to share accurate and accessible information even more significant.

The objective reasons for carrying out this study are mainly based on analysing the web accessibility of the World Health Organization (WHO) during the COVID-19 pandemic. Thus, the objective will be to determine whether the content offered to inform about the disease is prepared so that any person can access it, regardless of their technology (hardware, software, or network infrastructure), language, culture, or disability, whether physical or mental, as determined by the World Wide Web Consortium (W3C). This institution is an international community that aims to ensure that anyone can access the content offered by a website, promoting the social value it provides for all citizens [5]. A lack of equity of access to health information can generate a digital divide [6,7] and affect the ability to deal the disease [7]. Consequently, it represents a social determinant of health.

In addition, the elderly are an important group of citizens who benefit from web accessibility, since their skills are weaker as a result of age [8]. Bearing in mind that older people are considered one of the main groups at risk in this pandemic, it is especially important that they are the ones who have the most access to the content offered by information sources such as the WHO [9]. From the point of view of web accessibility, it should be emphasised how few studies have been carried out on international and official organisations with important social responsibilities such as the WHO, as they are more oriented towards the education sector such as universities and local corporations such as town councils [10,11,12,13,14]. Most of these studies do not focus on recent recommendations but only on some that have been detected in the area of education [15,16]; likewise, studies have shown that citizens’ dependence on the media is more intense in crisis situations [17]. However, traditional media and the Internet play different roles for people in these public health crises, and the Internet’s dependence on individuals is greater than that of traditional media [18]. Therefore, one of the major contributions of this research is based on the originality of the analysis of universally accessible public health risk communication in times of global pandemic. Likewise, the consideration that access to information is a health determinant is also relevant.

This research is structured in the following blocks. After the introduction, the theoretical framework shows the WHO as the main health agency and information reference in the COVID-19 pandemic. The methodology of the research is of an exploratory type, contemplating an analysis of the WHO’ page using the Wave tool and a manual review of each of the criteria of the Content Accessibility Guidelines. The results obtained reflect the level of accessibility of the WHO according to international standards.

The practical implications of this study highlight the need to raise awareness among health organisations such as the WHO about the importance of accessibility to their digital content in times of health crisis with the social objective of universal health information accessibility. The main contribution is the analysis of accessibility, taking particular account of elderly people, as one of the most vulnerable groups in this pandemic, and consequently, it provides practical proposals for addressing this challenge for health institutions.

## 2. Theoretical Framework

### 2.1. The World Health Organization and COVID-19

The WHO is an international organisation founded in 1948, and since then, the world has undergone great political and economic changes, not only from the point of view of health [19]. Its headquarters are located in Geneva, with 150 offices in different countries and six regional offices [20]. Any country that is a member of the United Nations can become a member of this organisation [21]. In order to become a member, countries must agree to its Constitution, which currently includes 194 member states [21]. Among the different activities addressed by this organisation, one of the main objectives is to provide universal health coverage by supporting integrated health services for citizens. They offer prevention, surveillance and a response to possible risks that may threaten citizen security, for example, in pandemics such as COVID-19 [22]. This underlines the importance of this organisation as the main source of information on this disease, which has become a pandemic caused by the coronavirus, the outbreak of which began in December 2019 in Wuhan, China, and has affected the whole world [23].

**Figure 1:**
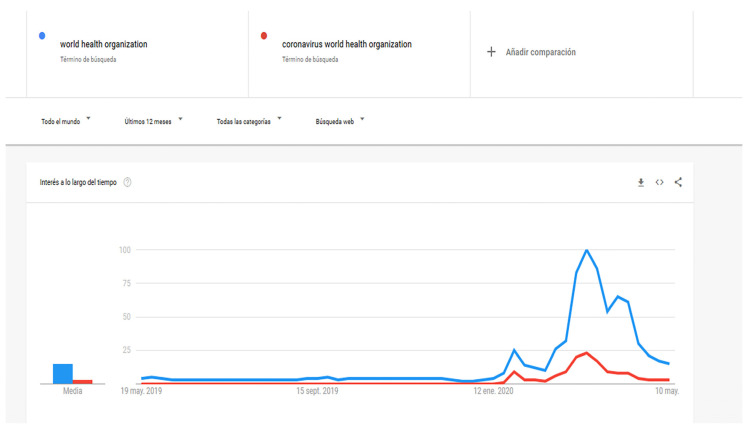
Google word search results for the keywords “world health organization” and “coronavirus world health organization” in the last 12 months. Source: prepared by the authors with the Google Trends tool (https://trends.google.es/).

The WHO acts as an agency that promotes accessibility in all its fields, including digital, and provides recommendations on its website [24]. For its part, the Spanish Association of Scientific Communication (AECC) [25] highlights the WHO as one of the main sources of consultation for the citizens of affected countries. In the United States, 53.1% of the population seeks health information on the Internet [26]. In order to offer as much information as possible on COVID-19, the WHO has created specific pages on prevention, symptoms and action protocols and even a solidarity page for donations to help research and detect the spread of the virus. These donations also go to UNICEF partners to support their work in communities that are the most vulnerable, such as children [27]. It is important to note that an outbreak such as COVID-19 causes important social consequences, affecting social distance in the most affected countries and generating more anxiety [28]. All of the above have resulted in an increase in Google searches for the keywords “world health organization” and “coronavirus world health organization” worldwide, which began to grow in early March, as can be seen in Figure 1.

**Figure 2:**
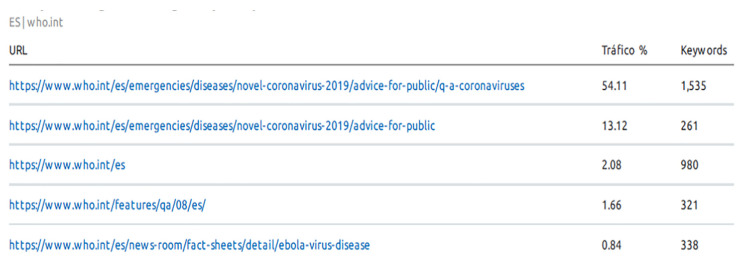
Organic searching of the World Health Organization’s (WHO’s) main web pages in the last 6 months.Source: based on Semrush tool (https://www.semrush.com/).

This search result confirms that the WHO has a social responsibility to provide quality content and information that is accessible to all types of people, since as the network evolves, different challenges are being addressed, resulting in a continuous need for relationships and trust [29]. Moreover, it is a way of ensuring equity, eliminating disparities and improving the health of all groups [30]. It is important to highlight the responsibility that the management of pandemics entails for the WHO, since depending on the type, it involves a problem of uncertainty when the end of the pandemic is declared, as occurred in the case of influenza A (H1N1) in 2009 [31]. Lamb-White [32] refers to the WHO’s commitment to improving communicable diseases through the International Health Regulations (IHR) to improve public health, and this would therefore help countries to strengthen their capacity to achieve this. Figure 2 shows the significant increase in visits, unique visitors and pages per visit in the last 6 months for the WHO website. Moreover, it is surprising that the average duration of visits has also increased, so it can be said that the WHO website has been and is a reference for consultation on public health on a global level, especially in times of pandemics such as COVID-19, as can be seen in the data. In addition, the semrush tool (semrush.com), comparing the WHO’s traffic data with those of the website of the European Centre for Disease Prevention and Control (the second most popular website after the WHO’s according to the European Parliament’s European Science-Media Hub [2]), shows that the WHO website visits increased from the end of February to April.

**Figure 3:**
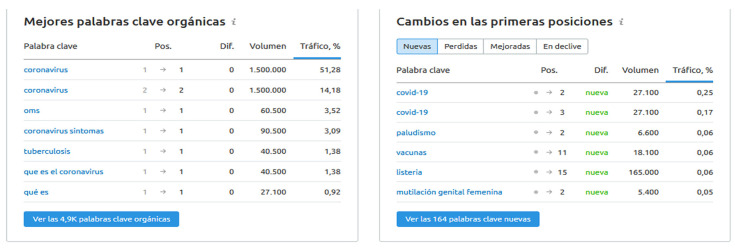
Organic searching for keywords on the WHO website in the last 6 months. Source: Based on Semrush tool (https://www.semrush.com/).

Figure 2 shows that the WHO pages related to the COVID-19 pandemic were the most visited in the last 6 months according to a study by the European Science-Media Hub of the European Parliament [2]. Specifically, this tool has shown that in countries such as Spain, the keyword “coronavirus” has increased organic traffic on the WHO website, accounting for more than 50% of organic web traffic, as can be seen in Figure 3, in addition to terms such as COVID-19 being incorporated in the top positions, showing that citizens have real public health concerns through the search for these keywords that are incorporated into the ranking of new searches related to the WHO.

Apart from the organic traffic referenced above, it should be noted that the WHO is making great communication efforts to reach all citizens through the paid searches of search engines such as Google, specifically through ads in different languages, depending on the search keyword.

### 2.2. Relevance of Communication and Web Accessibility in Official Organisations

People with disabilities have many difficulties in becoming independent as a result of the lack of commitment of the different public policies in force. A report by the Spanish Committee of Representatives of People with Disabilities (CERMI) states: “Universal accessibility is the great failure of public policies in our country” [33] (p. 528). This causes people with disabilities to encounter physical and technological barriers in their daily lives. Consequently, it suggests that institutions with competences could address this matter and pay attention to accessibility to minimise the digital divide [6], achieve health equity, and allow all groups to better face the disease [7]. According to WebAIM, the Internet is an opportunity for people who have some kind of disability, since it allows them to access information through diverse content quickly and by means of different devices and software, for example, screen readers for people with vision problems. However, these opportunities that the World Wide Web (WWW) should offer through websites are not sufficiently optimised and adapted to the different needs of citizens according to their disability [34].

According to WHO data, the aging of the population and the increase in chronic diseases are one of the main reasons for the increase in disability rates, which is about 15% for the world’s population [24]. In fact, age is a physical and social determinant directly correlated with health [30].

From a legal perspective, Directive (EU) 2016/2102 of the European Parliament and of the Council of 26 October 2016 on the accessibility of websites and mobile devices requires public sector bodies to comply with the requirements of the Web Content Accessibility Guidelines by specifying the European standard EN 301 549 V1.1.2 (2015-04). Subsequently, the versions EN 301 549 v2.1.2 (2018-08) and EN 301 549 V3.1.1 (2019-11) were approved, reflecting the changes requested by the European Commission in which the novelties of the Web Content Accessibility Guidelines (WCAG) 2.1 levels A and AA were presented, which are the requirements that web pages must currently comply with. Mobile applications must comply with this from 23 June 2021.

In times of crisis, communication through written messages is remembered more than those transmitted through other formats, so it must be not only accessible but also accurate so that it is understood by the majority of the population [35].

Studies have confirmed that because of the speed with which these types of diseases such as COVID-19 are transmitted, citizens and different countries need to increase their vigilance and prepare themselves through preventive responses [36]. Communication is particularly important in this regard, as is access to equal opportunities for all. However, if information tends to be complex and ambiguous in terms of the interpretations that citizens may make, situations of panic and anxiety may arise [37].

Exceptional crisis situations such as the COVID-19 pandemic generate greater attention or dependence on information, especially reliable and accurate information. In addition to this maxim, which can be contrasted with previous literature, Internet penetration makes it easy to access information and increases the level of information that each person has, which is why the following research questions are posed:

RQ1: Does the WHO make itself accessible to all groups of citizens according to accepted standards in the field of the Internet?

RQ2: What aspects of web content analysis can be improved, and which audiences are affected?

## 3. Materials and Methods

This research analyses the web accessibility of the WHO website based on the Web Content Accessibility Guidelines 2.1 in an exploratory way. The analysis was carried out during the COVID-19 pandemic in March–May 2020, coinciding with one of the world’s most popular periods for citizens to search for information (Figure 1).

The methodology was combined using a web accessibility evaluation tool and manual analysis carried out by an evaluator [38,39]. The tool used for accessibility evaluation was the Wave tool [40], developed by the WebAIM organisation. The website accessibility conformity assessment methodology (WCAG-EM) was used, which is considered in the Web Content Accessibility Guidelines 1.0 but is applicable to WCAG 2.1 [41]. As for the variables analysed, they belong to the Web Content Accessibility Guidelines (WCAG), which explain how to make content more accessible to developers and other professional profiles related to web accessibility authoring and evaluation tools, including mobile accessibility [42].

It should be recalled in historical retrospect that WCAG 1.0 was a recommendation in May 1999. It consists of a total of 14 guidelines and 65 priority 1, 2 and 3 checkpoints depending on the level of compliance [43]. WCAG 2.0 was recommended in December 2008. Unlike the previous ones, it is composed of 12 guidelines and four principles—Perceptible, Operable, Understandable and Robust—with 61 criteria for success [44]. However, the latest guidelines recommended in June 2018 are WCAG 2.1, with a total of 13 guidelines and 78 compliance criteria; in this case, the W3C has included 17 new criteria, maintaining the four principles mentioned above [45]. The Web Content Accessibility Guidelines present different conformance levels—A, AA and AAA [43,44,45,46]. In the case of WCAG 1.0, the levels depend on satisfying the priority levels 1 to 3; for example, it is determined that level A is met when all the priority 1 checkpoints are satisfied [43]. However, in WCAG 2.0 and 2.1, the levels do not refer to priorities 1 to 3; for example, it is determined that level A is met when all the level A compliance criteria are satisfied [45,46].

### 3.1. Case Study

In order to carry out a more in-depth analysis, six representative pages from the entire website were analysed (Table 1). The sample was selected by taking representative pages that allow the checking of each of the analysed criteria; for example, a page with forms must be checked to determine the compliance of the labels in the fields, or a page with tables must be checked to ensure that the conent is in an accessible form; additionally, videos must be checked for their audiovisual accessibility. The methodology (WCAG-EM) suggested by W3C [41] recommends selecting representative URLs for each criterion:

1. Home page: the main page of the website.
2. Standard page: a second-level reference page of the website that describes the structure of the website.
3. Page with tables: a page that shows content laid out using tables.
4. Page with forms: registration forms, application forms, information forms, etc.
5. Result of a search: the information necessary for the location of contents is extracted and checked by means of a keyword search; in this case of analysis, the word “COVID-19” is used as an example.
6. Page containing video: to analyse compliance with the guidelines in the case of videos.

Once the representative URLs of the rest of the WHO website were selected, as shown in Table 1, the compliance with each of the variables to WCAG 2.1 was analysed. These are shown in Table 2 and Table 3, divided into levels of compliance A or double A respectively:

After analysing each of the criteria, the results obtained were collected with the data analysis tool (Table 4); the variables of the WCAG 2.1 analysed are shown in the upper part of the table, facilitating manual data collection and the checking of compliance. The first step was to check if the success criterion could be applied to the analysed URL and how many times it was applied to (A). The second step was to check whether the success criterion was approved (B) or not (M).

The symbols have the following meanings (Table 4):

P: Pages analysed for each service

A: Pages to which the criterion applies

B: Pages that are correct according to the criterion

M: Pages that breach the criterion

As can be seen in Table 4, the total number of pages analysed (TP) was calculated, the correct (TB) and incorrect (TM) pages were counted, and as a result, a percentage was obtained of the correct WHO pages, obtaining an average that represents the percentage of web accessibility compliance of each page analysed (%B).

The formula is as follows: (%B) = (TB × 100/TP).

### 3.2. Other Tools Used to Complement the Manual Analysis

For the manual assessment phase, Table 5 details the tools that enabled the level of compliance to be checked according to the WCAG 2.1 guidelines on those points that required more in-depth review, apart from the Wave tool, in the first phase.

## 4. Results

The analysis shows that the WHO website is 60% compliant regarding web accessibility based on the pages analysed; however, at the double-A level, the figure is slightly less than a 50% level of compliance. From the point of view of the four principles (Appendix A, Table A1) that underpin the WCAG 2.1—Perceptible, Operable, Understandable and Robust—it can be seen that the principle that is most complied with on the WHO website is Understandable at both levels, with 64% and 61.5% compliance, respectively. Therefore, the WHO’s digital health information is readable and understandable based on this principle [47].

However, Robust is the worst performer at both the A and AA levels, at 50% and 0%, respectively. It is precisely this principle that focuses on adapting the content to user applications and providing technical aids [47].

The Perceivable principle, directly related to the alternative text of the images, although it does not present outstanding values at level A with respect to the rest, is the second principle that best meets the double-A conformity criteria, with regard to the size and contrast of the text, benefiting those with vision problems derived from both age and sensory disabilities [47].

Finally, it should be noted that the Operable principle shows significant differences upon comparison at both levels, worsening at the double-A level, and therefore, navigation aspects have to be improved [47].

Generally, the principles that are most closely adhered to are found in level A, so it is concluded that those in level AA are the ones that need to be improved for each compliance criterion analysed in the WCAG 2.1.

**Figure 4:**
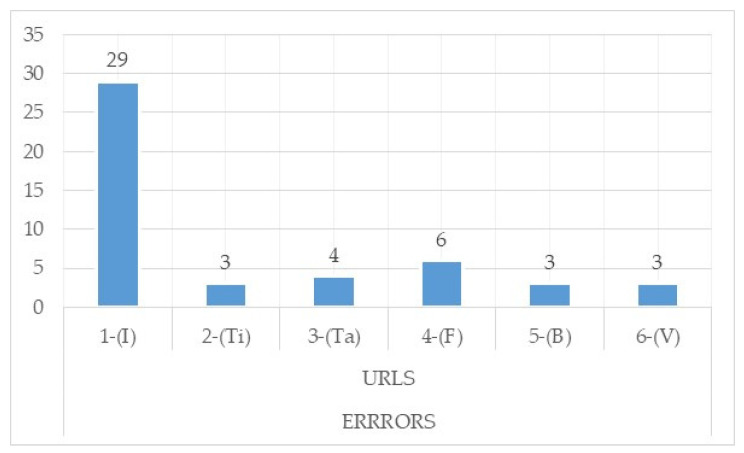
Total errors detected through Wave and checked manually.Source: own elaboration. Note:1-(I) URL 1-Start; 2-(Ti), URL 2-Page Type; 3-(Ta), URL 3-Page Tables; 4-(F), URL 4-Page with forms; 5-(B), URL 5-Finder; 6-(V), URL 6-Video.

With respect to the total number of errors detected by the Wave tool and later analysed manually, it is worth highlighting in Figure 4 that the home page is the one with the most errors, followed by the form page and the page with tables.

**Figure 5:**
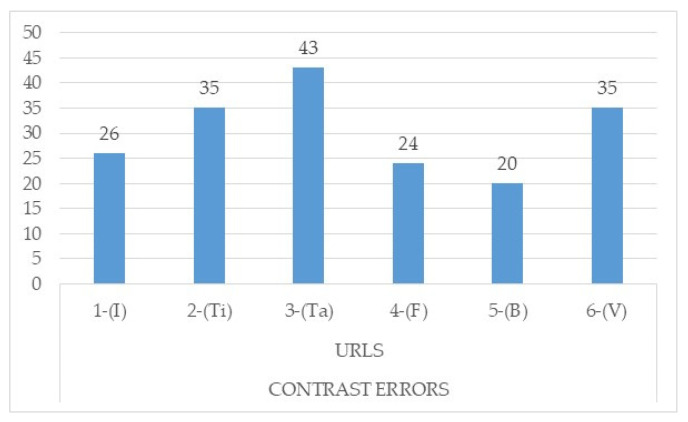
Total contrast errors detected through Wave and checked manually. Source: own elaboration. Note: 1-(I) URL 1-Start; 2-(Ti), URL 2-Page Type; 3-(Ta), URL 3-Page Tables; 4-(F), URL 4-Page with forms; 5-(B), URL 5-Finder; 6-(V), URL 6-Video.

**Figure 6:**
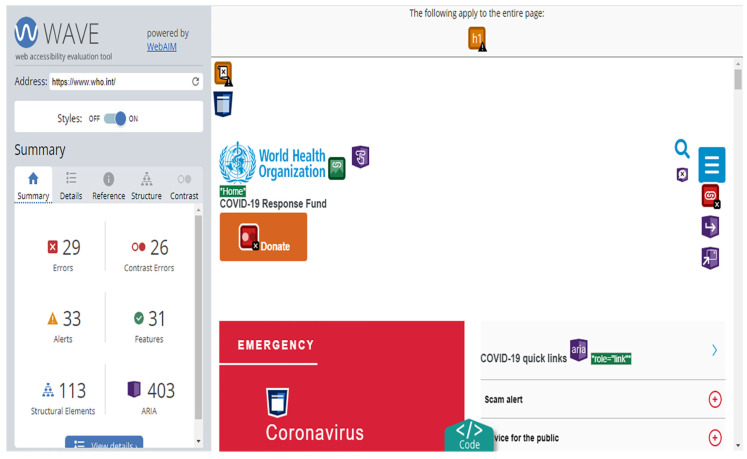
Example of Wave analysis on one of the pages analysed: https://www.who.int/home.

If one analyses it from the point of view of contrast errors, one will find that the page with the highest number of contrast errors is the table page, followed by the type page and the page with video (Figure 5 and Figure 6). Contrast errors are based on the fact that the visual presentation of the text and the images of the text must be sufficiently differentiated so that users with some type of visual disability can differentiate the text when reading, especially in the case of older age groups [47].

**Figure 7:**
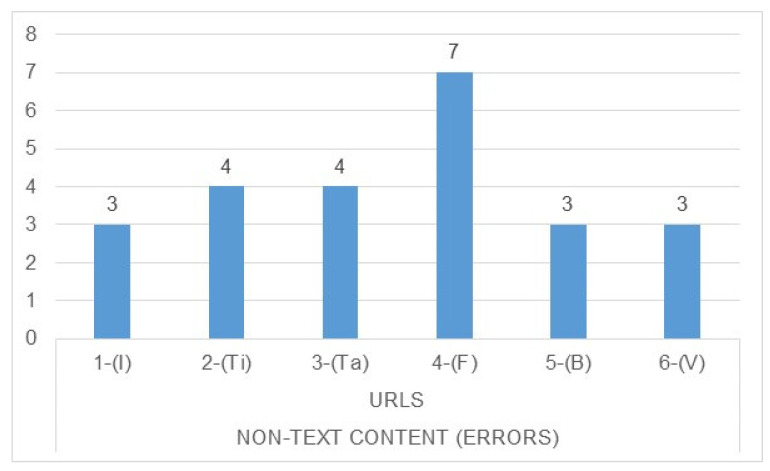
Total non-text content errors detected through Wave and checked manually. Source: own elaboration. Note: 1-(I) URL 1-Start; 2-(Ti), URL 2-Page Type; 3-(Ta), URL 3-Page Tables; 4-(F), URL 4-Page with forms; 5-(B), URL 5-Finder; 6-(V), URL 6-Video.

Regarding the non-text content errors detected, it can be seen that they also happen on all pages, especially form pages (Figure 7), so people who need a screen reader will be especially affected because there is no alternative text for the images.

**Figure 8:**
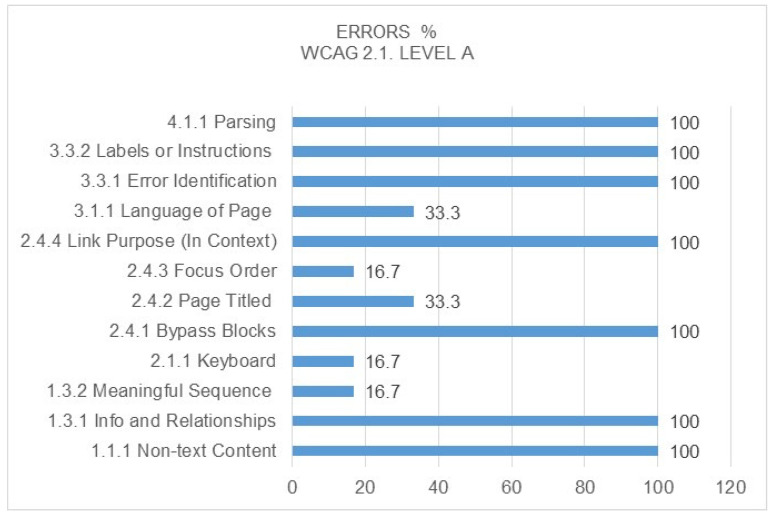
Percentage of errors detected at WCAG 2.1 Level A. Source: own elaboration.

**Figure 9:**
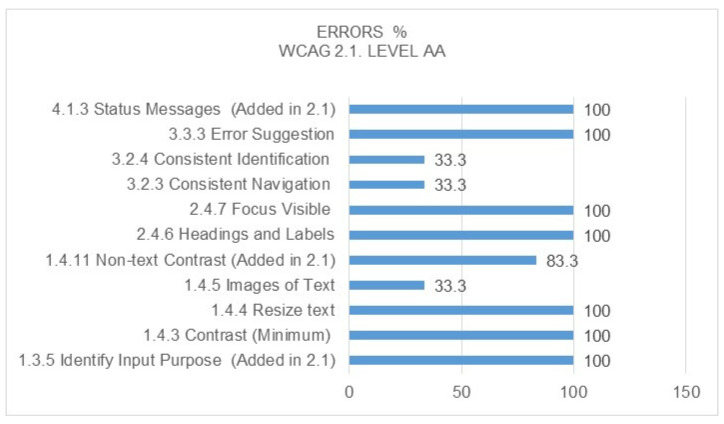
Percentage of errors detected at WCAG 2.1 Level AA. Source: own elaboration.

From the point of view of the type of errors detected, Figure 8 and Figure 9 show each of them in detail. It is worth noting from the comparison between the level A and double A errors that they coincide in both cases, and there are a total of seven success criteria with 100% error rates for the pages analysed. Of the total errors detected, the most significant are those referring to non-text content within Level A, as they are directly related to the alternative text of the image by means of the ALT tag, preventing screen readers from accessing the content by means of images for visually impaired citizens and, in the case of Level AA, the visible Focus, since if the user cannot clearly see where the keyboard tab is when browsing the page, it is difficult for them to conduct proper and understandable navigation through the content.

It is therefore determined that each of these errors detected requires a complete review that, in turn, contemplates alternative solutions based on guidelines established by the W3C to comply with the requirements set by the WCAG 2.1.

## 5. Discussion

As a main point of discussion, it should be noted that the WHO [24] states on its website that one of its objectives as an international organisation is based on improving universal accessibility to health services, both in Internet media and in physical media; however, its website does not show any kind of statement on accessibility with which they currently comply. It should be emphasised that the home page is one of the pages with the most errors; taking into account the fact that it is usually the first page that a user consults before proceeding to browse the rest of the web, it should be a principal target for improvement. In addition, the WHO recommends adopting digital media for health education [49], so it could be considered a special committer to digital media. As is seen in previous research work, assuming this responsibility is necessary to ensure health equity and to reduce the digital divide, which can affect the ability to face the disease for some population groups [6,7,30].

Considering the studies that have been carried out on citizens’ dependence on the media in times of public health crisis, most are based on analysing the differences between dependence on traditional media and that on the Internet [17,18], but they do not focus on analysing the information offered from the point of view of the accessibility of a particular agency and the social responsibility they have towards citizens facing a pandemic and seeking information.

Therefore, this study provides originality based on a specific case of accessibility in a health agency such as the WHO and provides points of improvement to make the content universal, at a crucial time of global pandemic, such as that presented by COVID-19. Furthermore, this article reinforces the conclusions reached by other studies in which it is highlighted that the population seeks information on public health mainly through the Internet [18,26]. From a technical point of view, more associated with web accessibility studies, it is worth mentioning that due to the recent approval of the WCAG 2.1, most studies have focused on the previous guidelines, so there is a shortage of studies based on the new guidelines, among which [15,16], focusing on the education sector, stand out.

## 6. Conclusions

Based on the results obtained, it is considered that the WHO is not accessible to all groups of citizens according to the Web Content Accessibility Guidelines 2.1, being less than 50% accessible at one of the levels analysed. It is concluded that many aspects need to be improved in order to make it fully accessible. One of the main online messages transmitted by the WHO [27] is “HEALTH FOR ALL”, and therefore, this research calls for “WEB ACCESSIBILITY FOR ALL” as the main aim and contribution to ensure that citizens have access to accurate, understandable and direct information; in short, there should be universal accessibility. It is also one of the overarching goals of the Healthy People 2020 initiative that specifically pays attention to achieving health equity and improving the health of all groups [50]. Hence, the concept of accessibility in times of crisis such as the COVID-19 pandemic is especially relevant, regarding the social value of the web.

Among the most notable errors are those concentrated largely within the principles Understandable and Perceivable, which shows that they are essential variables of communication with citizens, since they are directly related to content that is easy to understand in the first case and offer text alternatives for non-text content in the second case, especially for people with vision problems who use screen readers and even groups of elderly citizens who have vision problems as a result of physical aging. It is therefore determined that one of the aspects that most needs to be improved in terms of accessibility parameters is directly related to these two principles.

With regard to the limitations, it should be mentioned that the analysis could be completed with a heuristic study that would include manual checking as well as checking over time. On the other hand, comparison with other key information sources and the incorporation of WHO web users or those involved in this health crisis could offer a more complete vision of the phenomenon of web accessibility during a pandemic as well as the evaluation of the websites of other institutions.

With regard to future lines of research, since WCAG 2.1 must also be implemented in the mobile applications of public administrations, the analysis of the mobile applications of international organisations with social implications such as the WHO is proposed. It has also been considered as a future line of research to carry out user tests, a practice recommended by the W3C [51]. There are also different systems for groups with physical disabilities, where information can be collected from sources other than websites—for example, with bots—so it is proposed for these lines of research be considered in the future to make this research work more substantial.

The practical implications of this study are mainly based on the fact that international organisations with competence in the matter should review the structure and texts as well as everything related to the content in order to approach this challenge in an equitable way and provide the interested public with the same options of access to information. This study allows us to consider the accesibility of the WHO web resources with a special focus on elderly groups. The diagnosis performed will help health organisations to make decisions and to pay attention to critical points. The absence of text in images (non-text content) and errors in the HTML code (parsing) should be stressed. This study acts as a first attempt to analyse accessibility for the most representative health institution, the WHO.

This is the main social value that this research aims to convey, that the main sources of information—international organisations, whose responsibility for health is crucial in times of global pandemics such as the one we are experiencing—can be given solutions that provide greater visibility to the information.

## Figures and Tables

**Table 1:** List of pages analysed within the WHO website.

Urls Analysed | World Health Organization (WHO)	
1	https://www.who.int/
2	https://www.who.int/news-room/q-a-detail/q-a-coronaviruses
3	https://apps.who.int/gho/data/node.main-euro.RSUD760?lang=en&showonly=RSUD#
4	https://www.who.int/about/contact_form/es/
5	https://www.who.int/search?query=COVID-19&page=1&pagesize=10&sortdir=desc&sort=relevance&default=AND&f.Countries.size=100&f.Lang.filter=en&f.RegionalSites.size=100&f.Topics.size=100&f.contenttype.size=100&f.doctype.size=101&facet.field=RegionalSites&facet.field=Topics&facet.field=doctype&facet.field=Countries&facet.field=contenttype&facet.field=Lang&tune=true&tune.0=3&tune.1=2&tune.2=2&tune.3=3&tune.4=180&tune.5=75&cname=highlight-en&cname=emronew&cname=who&cname=euro&cname=afro&cname=amro&cname=pmnch&cname=searo&cname=workforcealliance&cname=wpro&f.RegionalSites.filter=Global&f.contenttype.filter=html
6	https://www.who.int/emergencies/diseases/novel-coronavirus-2019

Source: Own elaboration.

**Table 2:** Level A Web Content Accessibility Guidelines 2.1 analysis.

Principle	Guideline WCAG 2.1 (Success Criteria)	Checkpoints	Level
**1–Perceivable**	Guideline 1.1: Text Alternatives	1.1.1 Non-text Content	A
	Guideline 1.2: Time-based Media	1.2.1 Prerecorded Audio-only and Video-only	A
		1.2.2 Captions (Prerecorded)	A
		1.2.3 Audio Description or Media Alternative (Prerecorded)	A
	Guideline 1.3: Adaptable	1.3.1 Info and Relationships	A
		1.3.2 Meaningful Sequence	A
		1.3.3 Sensory Characteristics	A
	Guideline 1.4: Distinguishable	1.4.1 Use of Color	A
		1.4.2 Audio Control	A
**2–Operable**	Guideline 2.1: Keyboard Accessible	2.1.1 Keyboard	A
		2.1.2 No Keyboard Trap	A
		2.1.4 Character Key Shortcuts (Added in 2.1)	A
	Guideline 2.2: Enough Time	2.2.1 Timing Adjustable	A
		2.2.2 Pause, Stop, Hide	A
	Guideline 2.3: Seizures and Physical Reactions	2.3.1 Three Flashes or Below Threshold	A
	Guideline 2.4: Navigable	2.4.1 Bypass Blocks	A
		2.4.2 Page Titled	A
		2.4.3 Focus Order	A
		2.4.4 Link Purpose (In Context)	A
	Guideline 2.5: Input Modalities	2.5.1 Pointer Gestures (Added in 2.1)	A
		2.5.2 Pointer Cancellation (Added in 2.1)	A
		2.5.3 Label in Name (Added in 2.1)	A
		2.5.4 Motion Actuation (Added in 2.1)	A
**3–Understandable**	Guideline 3.1: Readable	3.1.1 Language of Page	A
	Guideline 3.2: Predictable	3.2.1 On Focus	A
		3.2.2 On Input	A
	Guideline 3.3: Input Assistance	3.3.1 Error Identification	A
		3.3.2 Labels or Instructions	A
**4–Robust**	Guideline 4.1: Compatible	4.1.1 Parsing	A
		4.1.2 Name, Role, Value	A

Source: Authors, based on information provided by the World Wide Web Consortium (W3C) [47].

**Table 3:** Level AA Web Content Accessibility Guidelines 2.1 analysis.

Principle	Guideline WCAG 2.1 (Success Criteria)	Checkpoints	Level
**1–Perceivable**	Guideline 1.2: Time-based Media	1.2.4 Captions (Live)	AA
		1.2.5 Audio Description (Prerecorded)	AA
	Guideline 1.3: Adaptable	1.3.4 Orientation (Added in 2.1)	AA
		1.3.5 Identify Input Purpose (Added in 2.1)	AA
	Guideline 1.4: Distinguishable	1.4.3 Contrast (Minimum)	AA
		1.4.4 Resize text	AA
		1.4.5 Images of Text	AA
		1.4.10 Reflow (Added in 2.1)	AA
		1.4.11 Non-text Contrast (Added in 2.1)	AA
		1.4.12 Text Spacing (Added in 2.1)	AA
		1.4.13 Content on Hover or Focus (Added in 2.1)	AA
**2–Operable**	Guideline 2.4: Navigable	2.4.5 Multiple Ways	AA
		2.4.6 Headings and Labels	AA
		2.4.7 Focus Visible	AA
**3–Understandable**	Guideline 3.1: Readable	3.1.2 Language of Parts	AA
	Guideline 3.2: Predictable	3.2.3 Consistent Navigation	AA
		3.2.4 Consistent Identification	AA
	Guideline 3.3: Input Assistance	3.3.3 Error Suggestion	AA
		3.3.4 Error Prevention (Legal, Financial, Data)	AA
**4–Robust**	Guideline 4.1: Compatible	4.1.3 Status Messages (Added in 2.1)	AA

Source: Authors, based on information provided by the W3C [47].

**Table 4:** Example data collection tool for Web Content Accessibility Guidelines (WCAG) 2.1 (A) and (AA) checkpoints.

		1.1.1			1.2.1; 1.2.2; 1.2.3; 1.4.2			1.3.1			1.3.2			1.3.3			1.4.1			2.1.1			2.1.2			2.1.4						
	P	A	B	M	A	B	M	A	B	M	A	B	M	A	B	M	A	B	M	A	B	M	A	B	M	A	B	M	TP	TB	TM	%B
WHO	6	0	0	0	0	0	0	0	0	0	0	0	0	0	0	0	0	0	0	0	0	0	0	0	0	0	0	0	0	0	0	0,00
**Total**	**6**	**0**	**0**	**0**	**0**	**0**	**0**	**0**	**0**	**0**	**0**	**0**	**0**	**0**	**0**	**0**	**0**	**0**	**0**	**0**	**0**	**0**	**0**	**0**	**0**	**0**	**0**	**0**	**0**	**0**	**0**	**0,00**

Source: author’s elaboration based on the infoaccessibility observatory of discapnet [48]. Note: 1.1.1 Non-text Content; 1.2.1 Prerecorded Audio-only and Video-only; 1.2.2 Captions; 1.2.3 Audio Description or Media Alternative; 1.4.2 Audio Control; 1.3.1 Info and Relationships; 1.3.2 Meaningful Sequence; 1.3.3 Sensory Characteristics; 1.4.1 Use of Color; 2.1.1 Keyboard; 2.1.2 No Keyboard Trap; 2.1.4 Character Key Shortcuts (Added in 2.1).

**Table 5:** Tools used to complement the wave tool and the manual review.

Tools	Description	Checkpoints	URL
Mozilla Firefox, Microsoft Internet Explorer, Google Chrome en Android, Safari en iOS	Use zoom out (Control + +) to increase text size and zoom in (control + −) to decrease	1.4.4 Resize text (AA)	https://www.w3.org/WAI/WCAG21/quickref/?versions=2.0#qr-visual-audio-contrast-scale
Google Mobile Friendly	Responsive Design	1.4.10 Reflow (AA)	https://search.google.com/test/mobile-friendly
Accessibility insights	Color Contrast Analyser	1.4.11 Non-text Contrast (AA)	https://accessibilityinsights.io/docs/en/web/overview
CSS Tool	It allows evaluating if the content is cut, overlapping or overflowing	1.4.12 Text Spacing (AA)	https://www.usableyaccesible.com/archivos/CSS_WCAG21_1_4_12.css
PEAT	Detection of epilepsy	2.3.1 Three Flashes or Below Threshold (A)	http://trace.wisc.edu/edu/peat
Validator (X) HTML de W3C	Validator Html	4.1.1 Parsing (A)	http://validator.w3.org

Source: own elaboration.

## Appendix A

**Table A1:** Established principles.

		%B-WCAG 2.1 (Level A)	%B-WCAG 2.1 (Level AA)
1	Perceivable	58.1	53.5
2	Operable	61.9	33.3
3	Understandable	64	61.5
4	Robust	50	0
	Weighted average	58.5	37.1

Source: own elaboration.
